# Model-Free Lens Distortion Correction Based on Phase Analysis of Fringe-Patterns

**DOI:** 10.3390/s21010209

**Published:** 2020-12-31

**Authors:** Jiawen Weng, Weishuai Zhou, Simin Ma, Pan Qi, Jingang Zhong

**Affiliations:** 1 Department of Applied Physics, South China Agricultural University, Guangzhou 510642, China; wengjw@scau.edu.cn (J.W.); 15037092@chu.edu.cn (S.M.); 2 Department of Optoelectronic Engineering, Jinan University, Guangzhou 510632, China; zws957020@stu2020.jnu.edu.cn; 3 Department of Electronics Engineering, Guangdong Communication Polytechnic, Guangzhou 510650, China; qiqipan@163.com; 4 Guangdong Provincial Key Laboratory of Optical Fiber Sensing and Communications, Guangzhou 510650, China

**Keywords:** distortion measurement, optical distortion, camera calibration, fringe analysis

## Abstract

The existing lens correction methods deal with the distortion correction by one or more specific image distortion models. However, distortion determination may fail when an unsuitable model is used. So, methods based on the distortion model would have some drawbacks. A model-free lens distortion correction based on the phase analysis of fringe-patterns is proposed in this paper. Firstly, the mathematical relationship of the distortion displacement and the modulated phase of the sinusoidal fringe-pattern are established in theory. By the phase demodulation analysis of the fringe-pattern, the distortion displacement map can be determined point by point for the whole distorted image. So, the image correction is achieved according to the distortion displacement map by a model-free approach. Furthermore, the distortion center, which is important in obtaining an optimal result, is measured by the instantaneous frequency distribution according to the character of distortion automatically. Numerical simulation and experiments performed by a wide-angle lens are carried out to validate the method.

## 1. Introduction

Camera lenses suffer from optical aberration; thus, the nonlinear distortion would be introduced into the captured image, especially for the lens with large field of view (FOV). Therefore, distortion correction is a significant problem in the analysis of digital images. The accurate distortion correction of lens is especially crucial for any computer vision task [1,2,3,4] that involves quantitative measurements in the geometric position determination, dimensional measurement, image recognition, and so on. Existing methods for distortion correction can be divided into two main categories: traditional vision measurement methods and learning-based methods.

For the traditional vision measurement methods, it falls into the following main types. One relies on a known measuring pattern [5,6,7], including straight lines, vanishing points, and a planar pattern. It estimates the parameters of the un-distortion function by a known pattern to achieve correction. It is simple and effective, but the distortion center in nonlinear optimization would lead to instabilities [8]. The second is the multiple view correction method [9,10,11], which utilizes the correspondences between points in different images to measure lens distortion parameters. It achieves auto-correction without any special pattern but requires a set of images captured from different views. The third is the plumb-line method [12,13,14,15], which makes the distortion parameter estimation by some distorted circular arcs. Accurate circular arcs detection is a very important aspect for the robustness and flexibility of this kind of method. Human supervision and some robust algorithms for circular arcs detection are developed. All the above-mentioned methods rely on some specific distortion models, such as the commonly used even-order polynomial model [16] proposed by Brown, division model [17] proposed by Fitzgibbon, and fisheye lens model [18]. The whole image achieves distortion correction by employing several characteristic points or lines for the analysis to find out the distortion parameters. It would have poor generalization abilities to other distortion models. Furthermore, it should be noted that all these distortion models achieve ideal circular symmetry.

For the learning-based methods, it can be divided into two kinds. The first one is the parameter-based method [19,20,21], which estimates the distortion parameters by using convolutional neural networks (CNNs) in terms of the single parameter division model or fisheye lens model. It would provide more accurate distortion parameters estimation. However, the networks are still trained by a synthesized distorted image dataset derived from some specific distortion models, which causes inferior results for other types of distortion models. Recently, some distortion correction methods demanding no specific distortion models by deep learning have been proposed. Liao et al. [22] introduced model-free distortion rectification by introducing a distortion distribution map. Li et al. [23] proposed bind geometric distortion correction by using the displacement field between distorted images and corrected images. For these methods, there are different types of distortion models involved into the synthesized distorted image dataset for training, and the distortion distribution map or displacement field are obtained according to these distortion models. It means that the distortion correction is still built on some distortion models, and the employed distortion models are circular symmetry. However, none of the existing mathematical distortion models can work well for all the real lenses with fabrication artifacts.

In addition, there are some model-free distortion correction methods. Munji [24] and Tecklenburg et al. [25] proposed a correction model based on finite elements. The remaining errors in the sensor space can be corrected by interpolation with the finite element. However, the interpolation effect will be reduced when the measured image points are not enough. Grompone von Gioi et al. [26] designed a model-free distortion correction method. It involves great computation and is time consuming, because the optimization algorithm and loop validation are used for high precision.

Fringe-pattern phase analysis [27,28,29], due to its advantage of highly automated full-field analysis, is widely used in various optical measurement technologies, such as interferometry, digital holography, moire fringe measurement, and so on. It is also used for lens distortion determination. Bräuer-Burchardt et al. [30] achieved lens distortion correction by Phasogrammetry. The experiment system and image processing are a bit complicated because both the projector lens distortion and camera lens distortion are involved. Li et al. [31] eliminated the projector lens distortion by employing the Levenberg–Marquardt algorithm for improving the measurement accuracy of fringe projection profilometry, where the lens distortion is described by a polynomial distortion model. We employed the phase analysis of one-dimensional measuring fringe-pattern and polynomial fitting for simple lens distortion elimination by assuming that the distortion is ideal circular symmetry [32].

In this paper, a model-free lens distortion correction based on the phase analysis of a fringe-pattern is proposed. Unlike the method in [32], the proposed method does not rely on circular symmetry assumption. In order to avoid using the distortion model and circular symmetry assumption, the proposed method uses two sets of directional orthogonal fringe patterns to obtain the distortion displacement of all points in the distorted image. Moreover, considering that the distortion center may be displaced from the image center by many factors, such as an offset of the lens center from the sensor center of the camera, a slight tilt of the sensor plane with respect to the lens, a misalignment of the individual components of a compound lens, and so on, the distortion center should be measured in accordance with specific conditions instead of being assumed as the image center directly. For the proposed method, the distortion center is measured by the instantaneous frequency distribution according to the character of distortion automatically. The theoretical description of distortion measurement based on the phase analysis of the sinusoidal fringe-pattern is introduced. The numerical simulation results and experimental results show the validity and advantages of the proposed method.

## 2. Principle of Model-Free Lens Distortion Correction Method

### 2.1. Lens Distortion

**Figure 1:**
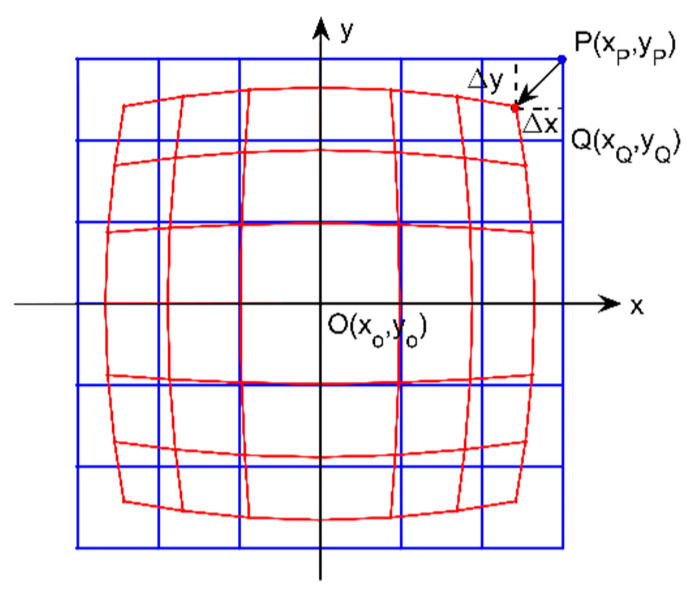
Distortion schematic diagram for the negative (barrel) distortion, where the blue and red lines are corresponding to the fringe-pattern stripes before and after distortion, respectively.

A simple grid chart of the negative (barrel) distortion, as shown in Figure 1, is employed to present the theoretical description of lens distortion simply and clearly. The blue lines and the red lines are corresponding to the lines before and after distortion, respectively. It is easy to find that the undistorted point P comes to the distorted point Q after distortion. According to the geometry shown in Figure 1, $\bar{PQ}$ is the distortion displacement Δr according to the distorted point Q with Δr>0 for the barrel distortion and Δr<0 for the pincushion distortion. So, the point $Q(x_{Q},y_{Q})$ should be corrected to the point $P(x_{P},y_{P})$, which satisfies:

(1) $$\left|\Delta r\right|=\sqrt{\Delta x^{2}+\Delta y^{2}}$$

with $\Delta x=x_{P}-x_{Q}$ and $\Delta y=y_{P}-y_{Q}$. For image distortion correction, the most important thing is to decide the distortion displacement Δr, i.e., Δx and Δy, at each point. Symbols denotation is given in nomenclature, as shown in Table 1.

### 2.2. Phase Analysis of Fringe-Pattern Measuring Template

**Figure 2:**
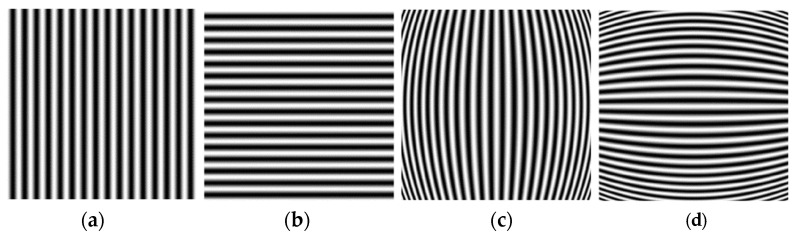
Sinusoidal fringe-patterns before and after barrel distortion. (**a**) Undistorted longitudinal fringe-pattern; (**b**) Undistorted transverse fringe-pattern; (**c**) Distorted longitudinal fringe-pattern; (**d**) Distorted transverse fringe-pattern.

Two sets of sinusoidal fringe-patterns parallel to the *y*-axis and *x*-axis of the display coordinate system, i.e., the longitudinal and transverse fringe-patterns, are employed as measuring templates for phase demodulation analysis to obtain the distortion displacement Δx and Δy, respectively. Figure 2 shows the sinusoidal fringe-patterns before and after barrel distortion, respectively. The undistorted longitudinal and transverse fringe-patterns are expressed as:

(2) $$\left\{ \begin{array}{l} I_{x}^{u}(x,y)=A+B\cos[2\pi f_{xo}x+\phi_{xo}]\\ I_{y}^{u}(x,y)=A+B\cos[2\pi f_{yo}y+\phi_{yo}] \end{array}\right..$$

The corresponding distorted fringe-patterns are:

(3) $$\left\{ \begin{array}{l} I_{x}^{d}(x,y)=A+B\cos[2\pi f_{xo}x+\phi_{x}(x,y)+\phi_{xo}]\\ I_{y}^{d}(x,y)=A+B\cos[2\pi f_{yo}y+\phi_{y}(x,y)+\phi_{yo}] \end{array}\right.$$

where A is the background intensity; B/A is the visibility of the fringe-pattern; $f_{xo}$ and $f_{yo}$ are the fundamental spatial frequency of the longitudinal and transverse sinusoidal fringe-pattern, respectively; $\phi_{x}(x,y)$ and $\phi_{y}(x,y)$ are the modulated phase caused by the lens distortion; $\phi_{xo}$ and $\phi_{yo}$ are the initial phase. By the analysis of the fringe-patterns, the modulated phase can be obtained point by point, so the distortion displacement Δx and Δy at each point is decided.

#### 2.2.1. Phase of Distorted Fringe-Pattens

The phase-shifting method [33], providing high precision point-to-point phase retrieval from fringe-patterns due to its best spatial localization merit, is employed for phase demodulation. The intensity distributions of the longitudinal and transverse sinusoidal fringe-patterns after distortion are:

(4) $$\left\{ \begin{array}{l} I_{x,n}^{d}(x,y)=A+B\cos\left[\varphi_{x}(x,y)+\frac{2\pi (n-1)}{N}\right]\\ I_{y,n}^{d}(x,y)=A+B\cos\left[\varphi_{y}(x,y)+\frac{2\pi (n-1)}{N}\right] \end{array}\right.$$

where $\varphi_{x}(x,y)=2\pi f_{xo}x+\phi_{x}(x,y)+\phi_{xo}$ and $\varphi_{y}(x,y)=2\pi f_{yo}y+\phi_{y}(x,y)+\phi_{yo}$ are the phase of longitudinal and transverse fringe-pattern, respectively; n=1,2,⋯,N and N=4. By employing the four-step phase-shifting method, the wrapped phase distribution can be acquired from the distorted fringe-patterns as:

(5) $$\left\{ \begin{array}{l} \varphi_{x}(x,y)=\arctan\left[\frac{I_{x,4}^{d}(x,y)-I_{x,2}^{d}(x,y)}{I_{x,1}^{d}(x,y)-I_{x,3}^{d}(x,y)}\right]\\ \varphi_{y}(x,y)=\arctan\left[\frac{I_{y,4}^{d}(x,y)-I_{y,2}^{d}(x,y)}{I_{y,1}^{d}(x,y)-I_{y,3}^{d}(x,y)}\right] \end{array}\right..$$

The calculated phase is wrapped in [−π,π] by arctangent calculation. So, the unwrapping algorithm [34] is performed, and the continuous phase distribution of the distorted fringe-pattern is obtained. More number of phase shifts would reduce the distortion of the cosine signal and improve the precision of phase demodulation.

#### 2.2.2. Modulated Phase Calculation by Distortion Center Detection

The modulated phases $\phi_{x}(x,y)$ and $\phi_{y}(x,y)$, which contain the distortion information, are calculated by subtracting the phase $\left(2\pi f_{xo}x+\phi_{x0}\right)$ and $\left(2\pi f_{yo}y+\phi_{y0}\right)$ from $\varphi_{x}(x,y)$ and $\varphi_{y}(x,y)$, respectively. So, in order to obtain the modulated phase, the fundamental spatial frequency $f_{xo}$ and $f_{yo}$ should be detected. According to the distortion character, there is no distortion at the distortion center. Therefore, the fundamental spatial frequency of the fringe-pattern is detected at the position of the distortion center. It should be noticed that the distortion center may be significantly displaced from the center of the image by many factors. For the proposed method, the distortion center can be measured by the instantaneous frequency distribution automatically. It is at the position where the minimum instantaneous frequency appears for the barrel distortion and the maximum instantaneous frequency appears for the pincushion distortion. So, we perform the partial derivative operation along the x and y direction of the phase $\varphi_{x}(x,y)$ and $\varphi_{y}(x,y)$ to get the instantaneous frequency $f_{x}$ and $f_{y}$ respectively as:

(6) $$\left\{ \begin{array}{l} f_{x}(x,y)=\frac{1}{2\pi }\frac{d}{dx}\varphi_{x}(x,y)\\ f_{y}(x,y)=\frac{1}{2\pi }\frac{d}{dy}\varphi_{y}(x,y) \end{array}\right..$$

By judging the variation trend of instantaneous frequency, the type of distortion could be determined. When the instantaneous frequency increases along the radial direction from the distortion center, it is the barrel distortion. Otherwise, it is the pincushion distortion. Then, by detecting the position of the minimum $f_{x}$ and $f_{y}$ along the x and y direction for the barrel distortion, or the position of the maximum $f_{x}$ and $f_{y}$ for the pincushion distortion, the distortion center can be decided as $\left(x_{0},y_{0}\right)$. So, the corresponding fundamental frequencies are determined at $\left(x_{0},y_{0}\right)$ as:

(7) $$\left\{ \begin{array}{l} f_{xo}={\frac{1}{2\pi }\frac{\partial \varphi_{x}(x,y)}{\partial x}|}_{\begin{array}{c} x=x_{0}\\ y=y_{0} \end{array}}\\ f_{yo}={\frac{1}{2\pi }\frac{\partial \varphi_{y}(x,y)}{\partial y}|}_{\begin{array}{c} x=x_{0}\\ y=y_{0} \end{array}} \end{array}\right..$$

According to $f_{xo}$ and $f_{yo}$, the phase distribution $2\pi f_{xo}x$ and $2\pi f_{yo}y$ can be calculated, and the modulated phase can be rewritten as $\Delta \varphi_{x}(x,y)$ and $\Delta \varphi_{y}(x,y)$:

(8) $$\left\{ \begin{array}{l} \Delta \varphi_{x}(x,y)=\varphi_{x}(x,y)-[2\pi f_{xo}(x-x_{0})+\varphi_{x}(x_{0},y_{0})]\\ \Delta \varphi_{y}(x,y)=\varphi_{y}(x,y)-[2\pi f_{yo}(x-x_{0})+\varphi_{y}(x_{0},y_{0})] \end{array}\right.$$

where $\varphi_{x}(x_{0},y_{0})$ and $\varphi_{y}(x_{0},y_{0})$ are the phase of the longitudinal and transverse sinusoidal fringe-patterns at the distortion center.

#### 2.2.3. Relationship of Modulated Phase and Distortion Displacement

The distortion displacement Δx(x,y) and Δy(x,y) are obtained by the modulated phase as:

(9) $$\left\{ \begin{array}{l} \Delta x(x,y)=\frac{\Delta \varphi_{x}(x,y)}{2\pi f_{xo}}\\ \Delta y(x,y)=\frac{\Delta \varphi_{y}(x,y)}{2\pi f_{yo}} \end{array}\right.\;\;\;.\;$$

Therefore, the measurement of the distortion displacement is transferred into the calculation of the modulated phase by the fringe-pattern analysis. In a word, the distortion displacement Δx(x,y) and Δy(x,y) can be measured point by point according to the phase demodulation analysis of the distorted sinusoidal fringe-patterns.

#### 2.2.4. Distortion Correction

The inverse mapping method with the bilinear interpolation is employed for the image distortion correction. It should be noticed that the distortion displacement obtained by Equation (9) corresponds to the points on the distorted fringe-pattern. So, we recalculate the distortion displacement $\Delta^{\prime }x$ and $\Delta^{\prime }y$, which correspond to the points on the corrected fringe-pattern. Firstly, we establish the discrete numerical correspondences of the new distortion displacement $\Delta^{\prime }x$ and $\Delta^{\prime }y$ with the points (x+Δx,y+Δy) on the corrected fringe-pattern. It should be noticed that the calculated coordinate value of the points (x+Δx,y+Δy) on the corrected fringe-pattern may not be integer. So, we calculate the distortion displacement $\Delta^{\prime }x(m,n)$ and $\Delta^{\prime }y(m,n)$ by performing the bicubic interpolation algorithm, where (m,n) is the integer coordinate value of the corrected point. Therefore, the distribution of the distortion displacement corresponding to the points on the corrected fringe-pattern is decided, and the image distortion correction can be achieved by adopting the inverse mapping method directly by:

(10) $$\left\{ \begin{array}{l} x_{d}^{m,n}=m-\Delta^{\prime }x(m,n)\\ y_{d}^{m,n}=n-\Delta^{\prime }y(m,n) \end{array}\right.$$

where $\left(x_{d}^{m,n},y_{d}^{m,n}\right)$ is the corresponding distorted point. Finally, the bilinear interpolation algorithm is employed for the image interpolation not only because of the simple and convenient calculation process but also due to its ability of overcoming the problem of gray-scale discontinuity.

## 3. Numerical Simulation

**Figure 3:**
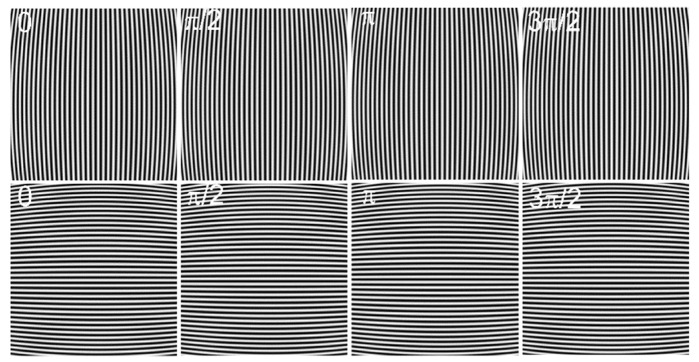
Simulated distorted fringe-patterns of size 512×512 pixels by a single parameter division model with a distortion parameter $\lambda \;=\;-1\;\times \;10^{-6}$ and distortion center (273,289). The longitudinal fringe-patterns with a phase shift of 0, π/2, π, 3π/2 are at the first row and the transverse fringe-patterns with a phase shift of 0, π/2, π, 3π/2 are at the second row.

Numerical simulation is performed to verify the validity of the method we introduced. Firstly, two sets of longitudinal and transverse sinusoidal fringe-patterns of 512 × 512 pixels with a phase shift of 0, π/2, π, 3π/2 are employed as measuring patterns. The spatial period of the undistorted fringe-pattern is 16 pixels. Then, the single parameter division model [17] with distortion parameter $\lambda \;=\;-1\;\times \;10^{-6}$, given by Equation (11), is employed to generate the barrel distorted fringe-patterns.

(11) $$r_{u}=\frac{r_{d}}{1+\lambda r_{d}{}^{2}}$$

where $r_{u}$ and $r_{d}$ are the Euclidean distance of the distorted and undistorted point to the distortion center, respectively. Moreover, the distortion center is shifted away from the image center (256,256) to (273,289). Figure 3 shows the corresponding distorted fringe-patterns, respectively. It should be noticed that the proposed method is model-free. The single parameter division model employed here, which can be replaced by any other distortion model, is just to generate a simulated distorted image.

The analysis process and the corresponding results can be described as follows.

**Figure 4:**
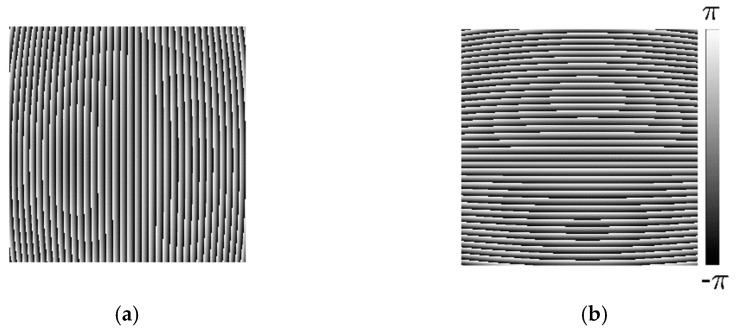
Wrapped phase. (**a**) Wrapped phase of distorted longitudinal fringe-pattern; (**b**) Wrapped phase of a distorted transverse fringe-pattern transverse.

Step 1: Employ the four-step phase-shifting method for the phase demodulation and perform the unwrapping algorithm to obtain the phase distribution of the distorted longitudinal and transverse fringe-patterns. Figure 4a,b are the corresponding wrapped phase.

**Figure 5:**
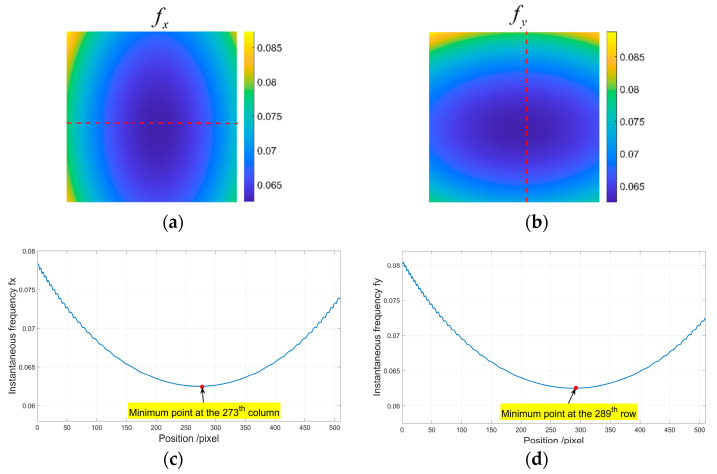
Instantaneous frequency. (**a**) $f_{x}$; (**b**) $f_{y}$; (**c**) $f_{x}$ at the 289th row with the minimum point at the 173th column; (**d**) $f_{y}$ at the 273th column with the minimum point at the 289th row.

Step 2: Calculate the instantaneous frequency $f_{x}$ and $f_{y}$. It is determined as barrel distortion because the instantaneous frequency increases along the radial direction from the distortion center. By detecting the minimum $f_{x}$ along the x direction, we can find that the distortion center is at the 273th column. Similarly, by detecting the minimum $f_{y}$ along the y direction, we can find that the distortion center is at the 289th row. So, the distortion center is at (273,289). Figure 5a,b $f_{x}$ and $f_{y}$, where the red dotted lines are at the 289th row and 273th column, respectively. Figure 5c,d are the corresponding distribution of $f_{x}$ at the 289th row and $f_{y}$ at the 273th column, respectively. The fundamental frequency is $f_{xo}=f_{yo}=0.0625$ and the phase $\varphi_{x}(x_{0},y_{0})$ and $\varphi_{y}(x_{0},y_{0})$ at the distortion center are obtained.

**Figure 6:**
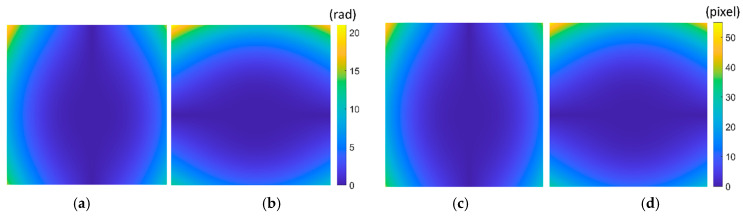
Modulated phase and distortion displacement. (**a**) $\Delta \varphi_{x}(x,y)$; (**b**) $\Delta \varphi_{y}(x,y)$; (**c**) Δx(x,y); (**d**) Δy(x,y).

Step3: Modulated phase calculation. According to Equation (8), the modulated phase $\Delta \varphi_{x}(x,y)$ and $\Delta \varphi_{y}(x,y)$ are obtained as shown in Figure 6a,b. So, the distortion displacement Δx(x,y) and Δy(x,y) are obtained point by point according to Equation (9), as shown in Figure 6c,d. The distortion displacement Δr can be obtained by Equation (1), and the maximum error of Δr is 0.24 pixels. In order to further validate the proposed method, distortion parameter estimation according to the single parameter division model is performed. The estimated distortion parameter is $\lambda \;=\;-0.9920\;\times \;10^{-6}$ with the relative error of 0.8%.

Step4: Distortion displacement map calculation. We establish the discrete numerical correspondences of the new distortion displacement $\Delta^{\prime }x$ and $\Delta^{\prime }y$ with the points (x+Δx,y+Δy) on the corrected fringe-pattern. Table 2 shows some of the distortion displacement of the points on the distorted fringe-pattern and the corresponding points on the corrected fringe-pattern. We find that the calculated coordinates of the points on the corrected fringe-pattern (x+Δx,y+Δy) are not integers. So, we calculate the distortion displacement $\Delta^{\prime }x(m,n)$ and $\Delta^{\prime }y(m,n)$ by performing the bicubic interpolation algorithm, where (m,n) is the integer coordinate value of the corrected point.

Step5: According to the distortion displacement map, image distortion correction can be performed by employing the inverse mapping with the bilinear interpolation directly.

**Figure 7:**
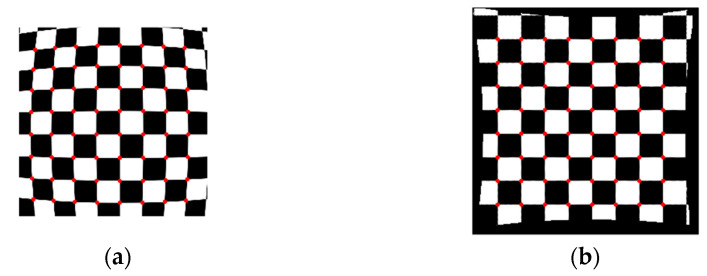
Analyzed results of checkerboard image, where the red points represent the corners. (**a**) Distorted checkerboard image with distortion parameter $\lambda \;=\;-1\;\times \;10^{-6}$ and distortion center (289,273); (**b**) Corrected image.

**Figure 8:**
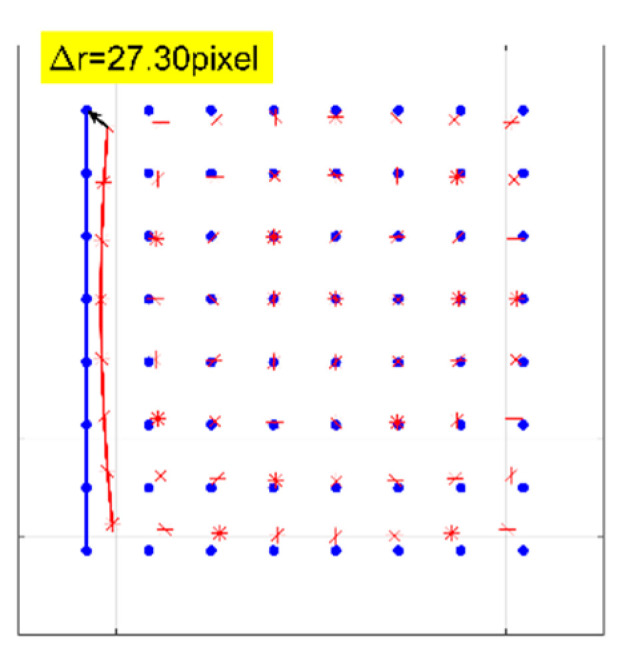
Corners image with the red asterisks representing the corners of the distorted checkerboard image and the blue points representing the corners of the corrected checkerboard image. The curvature radius of the red line is $2.2148\;\times \;10^{3}$ pixels, and that of the blue line is $1.4731\;\times \;10^{5}$ pixels.

A numerical simulation of the distorted checkerboard is performed. Figure 7a shows the distorted checkerboard image with the same distortion parameter of $\lambda =-1\;\times \;10^{-6}$ and distortion center (273,289). Figure 7b is the corrected result by the proposed method, where the red points represent the corners. Figure 8 is the corresponding corners image, where the red asterisks represent the corners of the distorted checkerboard image and the blue points represent the corners of the corrected checkerboard image. The distortion displacement at the left top point is Δr = 27.30 pixel. The curvature radius of the red line formed by the left points on the distorted checkerboard image is $2.2148\;\times \;10^{3}$ pixels, and that of the blue line corresponding to the corrected checkerboard image is $1.4731\;\times \;10^{5}$ pixels. It means that the circular arc is corrected to be straight.

## 4. Experiment and Results

**Figure 9:**
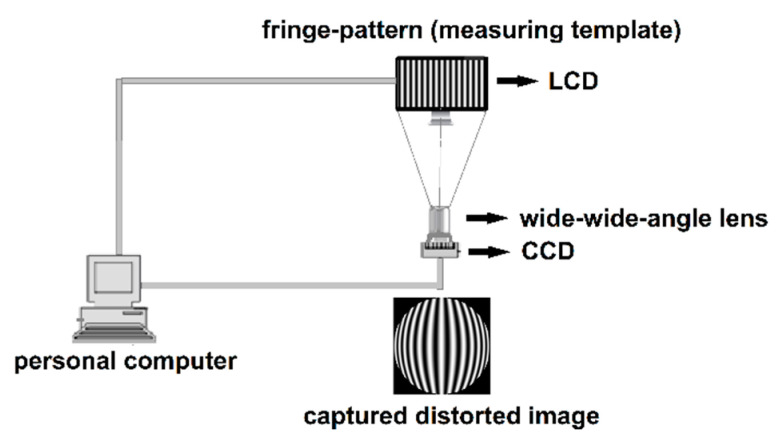
Experimental setup. Liquid crystal display (LCD): FunTV D49Y; wide-angle lens: Theia MY125M, FOV137^0^; charge-couple device (CCD) camera: PointGrey CM3-U3-50S5M-CS, 2048 × 2448 pixels, 3.45 μm pixel size.

**Figure 10:**
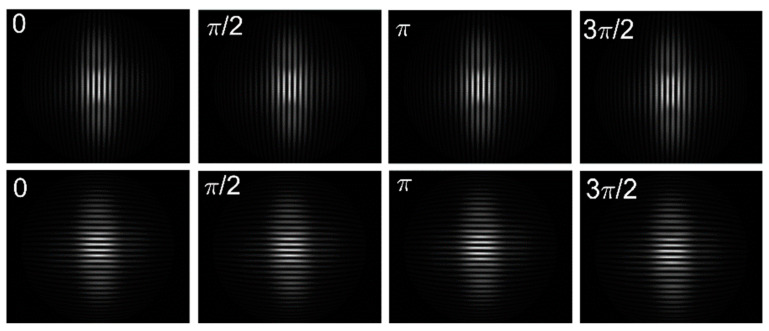
Distorted fringe-patterns of size 2048 × 2448 pixels. The longitudinal fringe-patterns with a phase shift of 0, π/2, π, 3π/2 are in the first row and the transverse with a phase shift of 0, π/2, π, 3π/2 are in the second row.

**Figure 11:**
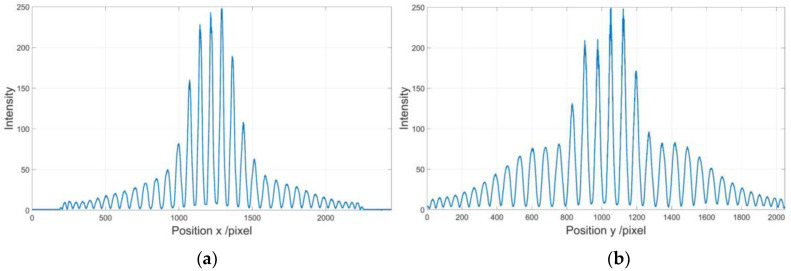
Intensity distribution of the distorted fringe-patters. (**a**) Intensity of the central row of the longitudinal fringe-pattern with a phase shift of π/2; (**b**) Intensity of the central column of the transverse fringe-pattern with a phase shift of π/2.

An experimental setup shown in Figure 9 is employed to perform distortion correction of a wide-angle lens. A flat-panel liquid crystal display (LCD) is used to display two sets of longitudinal and transverse sinusoidal fringe-patterns with phase shift of 0, π/2, π, 3π/2. The images of these fringe-patterns are captured by a charge-couple device (CCD) camera with a wide-angle lens. The LCD plane can be regarded as an ideal plane, and the optical axis of the camera is perpendicular to the LCD plane. The distorted fringe-patterns captured by the camera are shown in Figure 10. Figure 11a,b show the intensity distributions of the central row and column of the longitudinal and transverse fringe-patterns with a phase shift of π/2, respectively.

**Figure 12:**
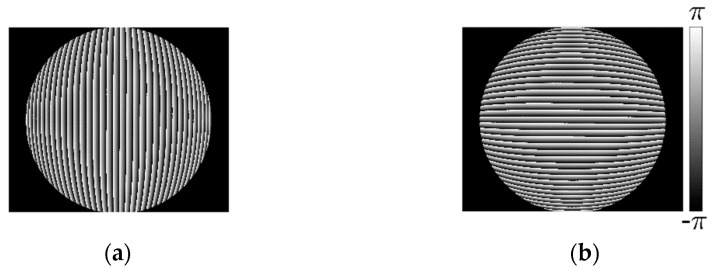
Wrapped phase. (**a**) Wrapped phase of distorted longitudinal fringe-pattern; (**b**) Wrapped phase of distorted transverse fringe-pattern transverse.

**Figure 13:**
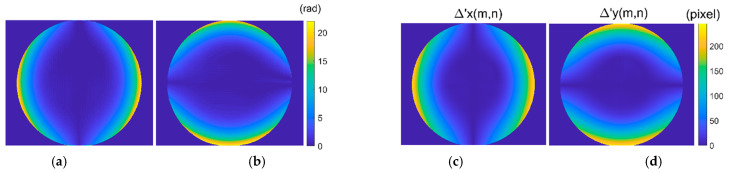
Modulated phase and distortion displacement map. (**a**) $\Delta \varphi_{x}(x,y)$ of size 2048×2448 pixels; (**b**) $\Delta \varphi_{y}(x,y)$ of size 2048×2448 pixels; (**c**) $\Delta^{\prime }x(m,n)$ of size 2496×2984 pixels; (**d**) $\Delta^{\prime }y(m,n)$ of size 2496×2984 pixels.

By performing the four-step phase-shifting analysis, the wrapped phases of the distorted longitudinal and transverse fringe-patterns are obtained as shown in Figure 12a,b. It shows that the phase of the distorted fringe-pattern can be demodulated well even when the intensity distribution of the fringe-patterns is low within some areas. The corresponding unwrapped phase can be obtained by performing the unwrapping algorithm. First, we perform the partial derivative operation of the phase of the distorted longitudinal and transverse fringe-pattern to get the instantaneous frequency $f_{x}$ and $f_{y}$, respectively. The type of distortion is determined as barrel distortion for the instantaneous frequency increases along the radial direction. Then, the distortion center is decided at (1224,1008) according to the distribution of the instantaneous frequency $f_{x}$ and $f_{y}$. Considering the fluctuation of the analyzed phase caused by the noise in the experiment, we perform numerical linear fitting to calculate the phase of the undistorted fringe-pattern by employing the central 25 points of the phase of the distorted longitudinal and transverse fringe-pattern respectively, instead of performing the calculation by the instantaneous frequency and phase at the distortion center. The points of $\left\{\varphi_{x}(x_{0}-12,y_{0}),\cdots \varphi_{x}(x_{0},y_{0}),\cdots \varphi_{x}(x_{0}+12,y_{0})\right\}$ and $\left\{\varphi_{y}(x_{0},y_{0}-12),\cdots \varphi_{y}(x_{0},y_{0}),\cdots \varphi_{y}(x_{0},y_{0}+12)\right\}$ are employed for calculation. The fundamental frequencies are $f_{xo}=f_{yo}=0.0133$. The modulated phase distribution $\Delta \varphi_{x}(x,y)$ and $\Delta \varphi_{y}(x,y)$ are obtained as shown in Figure 13a,b respectively. Figure 13c,d show the numerical distortion displacement map $\Delta^{\prime }x(m,n)$ and $\Delta^{\prime }y(m,n)$ of size 2496 × 2984 pixels. Finally, by employing the inverse mapping with the bilinear interpolation, the image distortion correction can be achieved.

**Figure 14:**
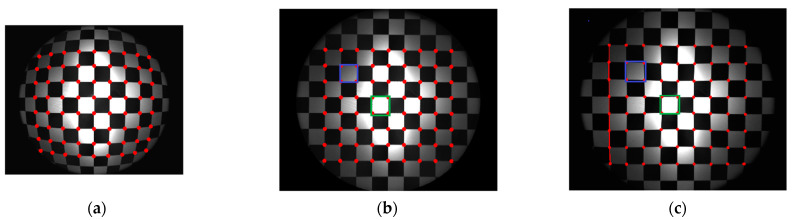
Experimental results of checkerboard images. (**a**) Distorted checkerboard image of size 2048×2448 pixels; (**b**) Corrected image of size 2496×2984 pixels with the red points representing the corners by the proposed model-free method. The pixel count of the square within the central green rectangular region is 49,033 pixels, and that of the square within the external blue rectangular region is 48,460 pixels. (**c**) Corrected image by plumb-line method. The curvature radius of the red line is $3.2821\;\times \;10^{4}$ pixels. The pixel count of the square within the central green rectangular region is 46988 pixels, and that of the square within the external blue rectangular region is 57,490 pixels.

**Figure 15:**
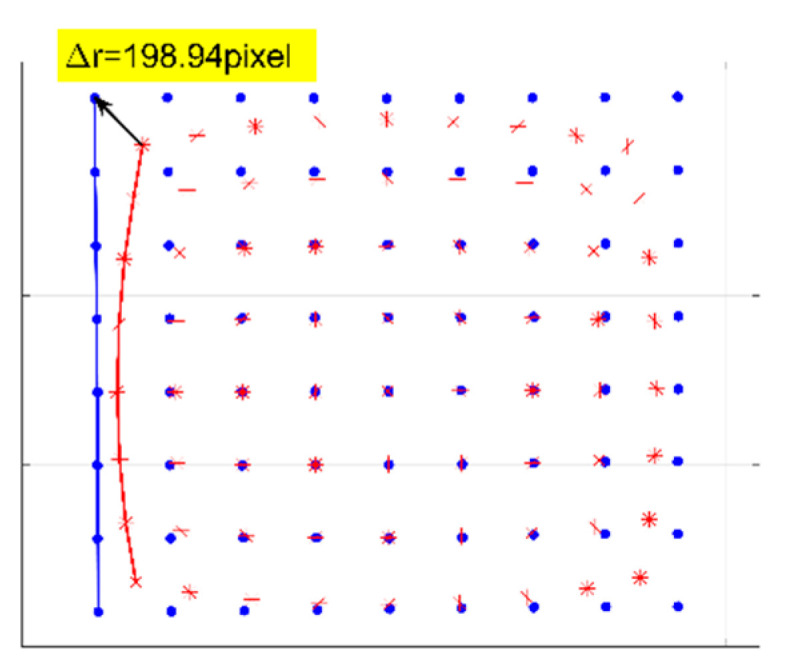
Corners image with the red asterisks representing the corners of the distorted checkerboard image and the blue points representing the corners of the corrected checkerboard image corresponding to those shown in Figure 14a,b. The curvature radius of the red line is $3.2457\;\times \;10^{3}$ pixels, and that of the blue line is $6.8656\;\times \;10^{4}$ pixels.

Figure 14 shows the experiment of checkerboard images, where Figure 14a is the distorted image and Figure 14b is the corrected image with the red points representing the corners by the proposed model-free method. Figure 15 is the corresponding corners image, where the red asterisks represent the corners of the distorted checkerboard image and the blue points represent the corners of the corrected checkerboard image. The distortion displacement at the left top point is Δr=198.94 pixels. The curvature radius of the red line formed by the left points on the distorted checkerboard image is $3.2457\;\times \;10^{3}$ pixels, and that of the blue line corresponding to the corrected checkerboard image is $6.8656\;\times \;10^{4}$ pixels. The larger the curvature radius, the closer the curve is to the straight line, i.e., the better the correction effect.

Firstly, we perform distortion correction by the plumb-line method with a single parameter division model [12] for comparison. The red line shown in Figure 15 is employed for the estimation of the distortion parameter. The estimated λ is $-2.1469\;\times \;10^{-7}$. According to Equation (11), the distortion displacement can be numerically calculated. The corrected image is shown in Figure 14c, where the red points represent the corners. The curvature radius of the red line is $3.2821\;\times \;10^{4}$ pixels, compared with the corresponding curvature radius of $6.8656\times 10^{4}$ pixels by the proposed method. Moreover, the square within the central region is not the same size as the square at the external region in the corrected image as shown in Figure 14c. We select two white squares at the central and external region to show the difference. The pixel count of the square within the central green rectangular region is 46,988 pixels compared that of the square within the external blue rectangular region being 57,490 pixels. By the proposed method, the pixel counts of these two corresponding squares are 49,033 and 48,460 pixels as shown in Figure 14b. It means that the estimated distortion lambda of the single parameter division model by this characteristic circular arc does not fit for the whole image correction.

On the other hand, in order to make comparison with the method employing some distortion models, we employ the method in [32] for distortion displacement detection. We take the distortion center point as the origin of the coordinate and perform the numerical curve fitting according to the discrete distortion displacement from the 1224th to the 2556th point at the 1008th row by three different distortion models, which are an even-order polynomial model with one and two distortion parameters and single parameter division model. The even-order polynomial model [16] is described as:

(12) $$r_{u}=r_{d}(1+\lambda_{1}r_{d}{}^{2}+\lambda_{2}r_{d}{}^{4}+\lambda_{3}r_{d}{}^{6}+\cdots ).$$

**Figure 16:**
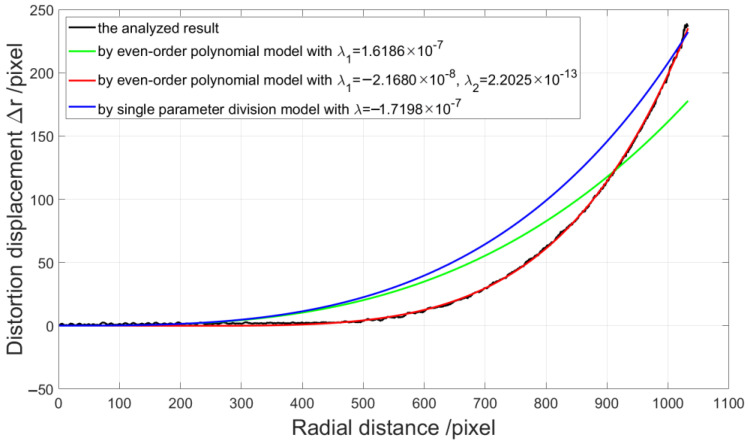
Radial distortion displacement and curve fitting results.

Figure 16 shows the curve fitting results, where the black line is the analyzed radial distortion displacement, the green line is the curve fitting result of the even-order polynomial model with $\left\{\lambda_{1}=1.6186\times 10^{-7}\right\}$, the red line is that of the even-order polynomial model with $\left\{\lambda_{1}=-2.1680\times 10^{-8},\lambda_{2}=2.2025\times 10^{-13}\right\}$, and the blue line is that of the single parameter division model with $\left\{\lambda =-1.7198\times 10^{-7}\right\}$. We can find that the fitting result by the even-order polynomial model with two distortion parameters is better.

**Figure 17:**
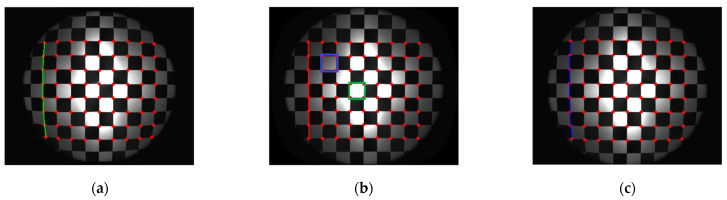
Experimental results of checkerboard images. (**a**) Corrected image by a one-parameter even-order polynomial model with a green line curvature radius of $6.9263\times 10^{3}$ pixels. (**b**) Corrected image by a two-parameter even-order polynomial model with a red line curvature radius of $2.3763\times 10^{4}$ pixels. The pixel count of the square within the central green rectangular region is 48,094 pixels, and that of the square within the external blue rectangular region is 48,694 pixels. (**c**) Corrected image by a single parameter division model with a blue line curvature radius of $1.1747\times 10^{4}$ pixels.

Figure 17 shows the corresponding correction results of the checkerboard image respectively, where Figure 17a,b are by the even-order polynomial model with one and two distortion parameters, respectively, and Figure 17c is by the single parameter division model. The curvature radius of the line formed by the left points on the corrected checkerboard image is $6.9263\times 10^{3}$ pixels, $2.3763\times 10^{4}$ pixels, and $1.1747\times 10^{4}$ pixels respectively compared with the corresponding curvature radius of $6.8656\times 10^{4}$ pixels by the proposed method. The pixel counts of the above-mentioned squares at the central and external regions in the corrected image by the even-order polynomial model with two distortion parameters are 48,094 and 48,694 pixels, as shown in Figure 17b. We can find that the curve fitting results rely on the distortion model greatly. Distortion determination may fail using an unsuitable model or by estimation of too few distortion parameters. However, the more distortion parameters there are, the more complicated the solution of the reverse process.

**Figure 18:**
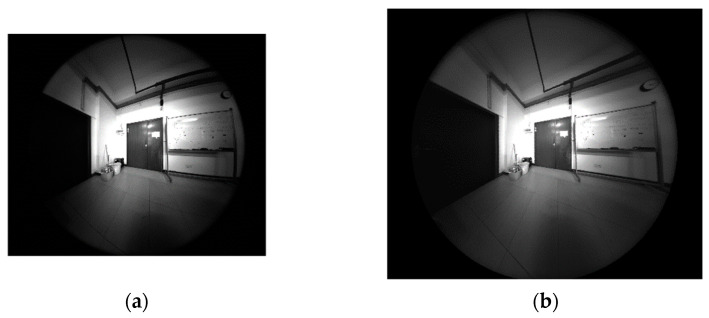
Experimental results of the indoor scene. (**a**) Distorted image of size 2048 × 2448 pixels; (**b**) Corrected image of size 2496 × 2984 pixels.

**Figure 19:**
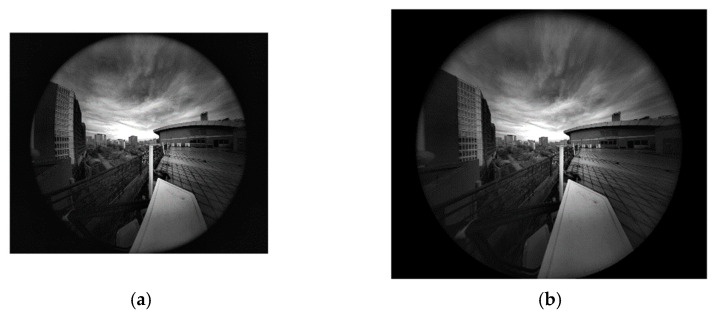
Experimental results of the outdoor scene. (**a**) Distorted image of size 2048×2448 pixels; (**b**) Corrected image of size 2496×2984 pixels.

Furthermore, the indoor and outdoor scenes are also employed for the experiment to show the practicality of the proposed method. Figure 18 and Figure 19 show the corresponding distorted and corrected image, respectively. The experimental results show that the distorted images achieve distortion correction effectively by the proposed model-free method.

## 5. Discussion

Experimental correction results of the checkerboard image by different methods are given for the comparison with the proposed model-free correction method. The original straight line is distorted into a circular arc with a curvature radius of $3.2457\times 10^{3}$ pixels, as shown in Figure 15. By the plumb-line method with the single parameter division model, the phase analysis method by the even-order polynomial model with one and two distortion parameters and the single parameter division model, and the proposed model-free method, the curvature radiuses of the corresponding corrected lines on the corrected checkerboard images are $3.2821\times 10^{4}$, $6.9263\times 10^{3}$,$2.3763\times 10^{4}$,$1.1747\times 10^{4}$, and $6.8656\times 10^{4}$ pixels, respectively. The circular arc is corrected to be straighter by the proposed method. It means that the proposed method provides a superior result, and it shows that distortion determination may fail using an unsuitable model firstly. Moreover, from the comparison of the curvature radius of the corresponding corrected lines, it seems that the corrected result of the plumb-line method is better than that by the phase analysis method by the even-order polynomial model with two distortion parameters. The reason for this is that the distortion parameter estimation by the plumb-line method is performed by the distorted circular arc at this position. However, for the corrected checkerboard image by the plumb-line method, the square within the central region of size 46,988 pixels is not the same as the square at the external region of size 57,490 pixels. It means that the estimated distortion parameter does not fit for the whole image correction. Therefore, according to the analysis result, we should take more characteristic points and lines or some other more complicated algorithm or distortion models into account. However, the more distortion parameters there are, the more complicated the solution of the reverse process. For the proposed model-free method, all points of the distorted fringe-pattern are employed for the establishment of the distortion displacement map, which demands none of the distortion model. So, the image achieve distortion correction point by point with a more effective and satisfactory result.

In the experiment of distortion displacement measurement, the errors caused by the nonideal LCD plane and imperfect perpendicular arrangement of the optical axis of camera and the LCD plane should be considered.

## 6. Conclusions

In this paper, a model-free lens distortion correction method based on the distortion displacement map by the phase analysis of fringe-patterns is proposed. For the image distortion correction, the most important thing is to decide the distortion displacement. So, the mathematical relationship of the distortion displacement and the modulated phase of the fringe-pattern is established in theory firstly. Then, two sets of longitudinal and transverse fringe-patterns are employed for phase demodulation analysis to obtain the distortion displacement Δx and Δy respectively by the phase-shifting method. The distortion displacement map can be determined point by point for the whole distorted image to achieve distortion correction. It would be effective even when the circular symmetry condition is not satisfied. Moreover, it detects the radial distortion type and the distortion center automatically according to the instantaneous frequency, which is important in obtaining optimal result. The correction results of the numerical simulation, experiments, and comparison show the effectiveness and superiority of the proposed method.

There are some prospects of our further works. Firstly, the relationship of the modulated phase and the distortion displacement described by the proposed method would still hold for the mix distortion with the radial and tangential type. However, if the tangential distortion is severe, the distortion center would not be the corresponding position where the minimum or maximum instantaneous frequency appears. So, how to decide the distortion center automatically in this case should be considered. Secondly, the optimal frequency of measuring fringe-patterns for accurate modulated phase analysis should be considered. Thirdly, the application of the proposed method should be implemented.

## Figures and Tables

**Table 1:** Nomenclature.

Term	Description
$I_{x}^{u}$; $I_{y}^{u}$	undistorted longitudinal and transverse fringe-patterns
$I_{x}^{d}$; $I_{y}^{d}$	distorted longitudinal and transverse fringe-patterns
$I_{x,n}^{d}$; $I_{y,n}^{d}$	distorted longitudinal and transverse fringe-patterns with phase shift
$\varphi_{x}$; $\varphi_{y}$	phase of distorted longitudinal and transverse fringe-patterns
$\phi_{x},\;\Delta \varphi_{x}$; $\phi_{y},\;\Delta \varphi_{y}$	modulated phase of distorted longitudinal and transverse fringe-patterns
$\phi_{xo}$; $\phi_{yo}$	initial phase of distorted longitudinal and transverse fringe-patterns
$f_{x}$; $f_{y}$	instantaneous frequency of distorted longitudinal and transverse fringe-patterns
$f_{xo}$; $f_{yo}$	fundamental frequency of distorted longitudinal and transverse fringe-patterns
Δx; Δy	distortion displacement of points on distorted fringe-pattern
$\Delta x^{\prime }$; $\Delta y^{\prime }$	distortion displacement of points on corrected fringe-pattern
$\left(x_{d}^{m,n},y_{d}^{m,n}\right)$	point on distorted image corresponding to point on corrected image with (m, n) being integer

**Table 2:** Some calculated distortion displacement (pixel).

distortion displacement of points on distorted fringe-pattern	Δx(x,y)	10.92 (197,121)	11.06 (198,121)	11.20 (199,121)
	Δy(x,y)	6.71 (197,121)	6.76 (198,121)	6.81 (199,121)
distortion displacement of points on corrected fringe-pattern	$\Delta^{\prime }x(x+\Delta x,y+\Delta y)$	10.92 (207.92,127.71)	11.06 (209.06,127.76)	11.20 (210.20,127.81)
	$\Delta^{\prime }y(x+\Delta x,y+\Delta y)$	6.71 (207.92,127.71)	6.76 (209.06,127.76)	6.81 (210.20,127.81)
distortion displacement of integer points on corrected fringe-pattern	$\Delta^{\prime }x(m,n)$	10.94 (208,128)	11.06 (209,128)	11.19 (210,128)
	$\Delta^{\prime }y(m,n)$	6.73 (208,128)	6.78 (209,128)	6.82 (210,128)

## Data Availability

Data sharing not applicable.
